# A Novel Feature-Selection Algorithm in IoT Networks for Intrusion Detection

**DOI:** 10.3390/s23198153

**Published:** 2023-09-28

**Authors:** Anjum Nazir, Zulfiqar Memon, Touseef Sadiq, Hameedur Rahman, Inam Ullah Khan

**Affiliations:** 1 Department of Computer Science, National University of Computer and Emerging Sciences (NUCES—FAST), Karachi 75123, Pakistan; annazir@gmail.com (A.N.); zulfiqar.memon@nu.edu.pk (Z.M.); 2 Centre for Artificial Intelligence Research, Department of Information and Communication Technology, University of Agder, Jon Lilletuns vei 9, 4879 Grimstad, Norway; 3 Department of Computer Games Development, Faculty of Computing & AI, Air University, E9, Islamabad 44400, Pakistan; hameed.rahman@mail.au.edu.pk; 4 Department of Electronic Engineering, School of Engineering & Applied Sciences (SEAS), Isra University, Islamabad Campus, Islamabad 44400, Pakistan; inamullahkhan05@gmail.com

**Keywords:** IoT, intrusions, machine learning, feature selection

## Abstract

The Internet of Things (IoT) and network-enabled smart devices are crucial to the digitally interconnected society of the present day. However, the increased reliance on IoT devices increases their susceptibility to malicious activities within network traffic, posing significant challenges to cybersecurity. As a result, both system administrators and end users are negatively affected by these malevolent behaviours. Intrusion-detection systems (IDSs) are commonly deployed as a cyber attack defence mechanism to mitigate such risks. IDS plays a crucial role in identifying and preventing cyber hazards within IoT networks. However, the development of an efficient and rapid IDS system for the detection of cyber attacks remains a challenging area of research. Moreover, IDS datasets contain multiple features, so the implementation of feature selection (FS) is required to design an effective and timely IDS. The FS procedure seeks to eliminate irrelevant and redundant features from large IDS datasets, thereby improving the intrusion-detection system’s overall performance. In this paper, we propose a hybrid wrapper-based feature-selection algorithm that is based on the concepts of the Cellular Automata (CA) engine and Tabu Search (TS)-based aspiration criteria. We used a Random Forest (RF) ensemble learning classifier to evaluate the fitness of the selected features. The proposed algorithm, CAT-S, was tested on the TON_IoT dataset. The simulation results demonstrate that the proposed algorithm, CAT-S, enhances classification accuracy while simultaneously reducing the number of features and the false positive rate.

## 1. Introduction

The Internet of Things (IoT) has emerged as a transformative technology, connecting a vast array of physical objects to the Internet. It can be defined as a network of physical objects that can sense (some physical phenomenon), communicate, and/or interact with the environment by generating some response [1]. This network of interconnected devices promises unprecedented convenience and efficiency across diverse domains, from smart homes and healthcare to industrial automation and transportation. However, this proliferation of IoT devices also presents significant security challenges.

In a recent study, Lee I. et al. [2] discussed how the attacks on IoT networks have rapidly and widely increased in the last few years. Organisations and individuals are facing a wide array of challenges. According to Gartner, 20% of organisations have already observed cyber attacks on IoT devices in the past three years [3]. The Internet Security Threat Report (ISTR) published by Symantec in the year 2019 [4] states that in 2017 alone, there was a 600% increase in attacks against IoT devices. These studies and statistics show that attacks on IoT networks are increasing at an alarming rate and that there is a dire need to address them.

Earlier, Louvieris et al. [5] conducted a detailed study in which they pointed out several prevalent reasons that have caused the high growth in cyber attacks. Below, we have presented a few notable causes discussed in the literature that have boosted growth rate of attacks against IoT devices or IoT-based networks/systems.

- IoT systems are relatively easy to attack compared to normal networks because IoT vendors focus more on device cost, usability, dimensions, etc., as compared to the security [6].
- Nearly all IoT-enabled devices communicate insecurely over unencrypted channels. This is a critical vulnerability that can easily disclose sensitive information to unauthorised users [7,8].
- A large number of IoT devices are exposed to medium- to high-risk vulnerabilities [9].
- Common Vulnerabilities and Exposure (CVE) [9] data for IoT devices showed that several devices, such as endoscopic cameras and blood pressure monitoring devices, use vulnerable operating systems or software packages, leaving the whole infrastructure vulnerable.
- IoT networks generally use lightweight communication protocols and weak security standards/techniques; therefore, they are easily exploitable [10].

Organisations use various security measures, such as firewalls, antivirus software, and intrusion detection and prevention systems, to protect against cyber attacks. However, these solutions often fall short in providing sufficient protection against evolving threats. As a result, organisations risk damage to their credibility, reputation, revenue, and customer base.

Antivirus software, typically installed on end devices, aims to protect against malware by identifying malicious signatures or patterns [11]. Its main limitation lies in its dependency on up-to-date signature databases, as it cannot detect or block files with unknown signatures.

Similarly, the majority of common firewalls can only filter network traffic based on source/target IP addresses or port numbers/services [12,13]. In contrast, modern firewalls like Next-Generation Firewalls (NGFWs) often offer Deep Packet Inspection (DPI), enabling them to scrutinise the payload within packets for signs of abnormalities or malicious activities [14]. Nevertheless, they frequently rely on signature-based techniques.

**Figure 1:**
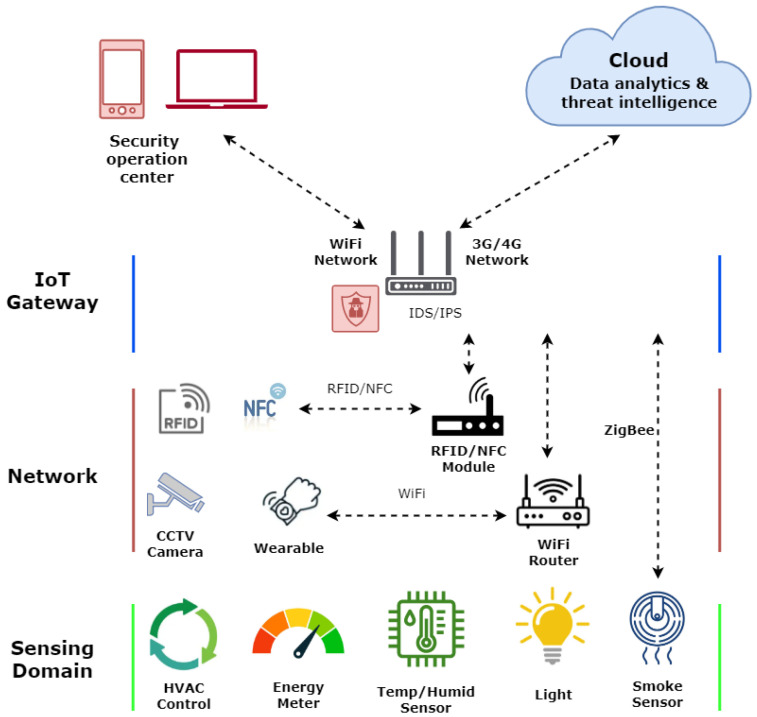
General features of a secure IoT network. An IDS/IPS sensor deployed at the gateway can detect and stop intrusions.

In contrast, Intrusion Detection and Prevention Systems (IDSs/IPSs) have the capability to identify and thwart network intrusions through either signature or anomaly detection methods. Signature-based IDSs/IPSs rely on the discussed signatures, whereas anomaly-based IDSs/IPSs employ various statistical techniques to identify intrusions within network traffic. As a result, anomaly-based IDSs can detect novel attacks that signature-based IDSs may overlook. In Figure 1, we present how an IDS/IPS can be deployed in IoT networks that can detect and block malicious activities. Besides its capability to detect unknown intrusions, anomaly-based IDS has the drawback of a high false positive rate and introduces additional challenges such as the need for network training and the involvement of domain experts to distinguish between false positives and true positives.

Besides several shortcomings, the literature shows that researchers have mainly focused on anomaly-based detection approaches due to their tendency to detect novel attacks [15,16]. In order to develop an efficient IDS capable of detecting novel attacks with high accuracy and low false positives, machine learning (ML) techniques have been intensively studied by researchers. Machine learning is an evolving branch of Artificial Intelligence (AI) that employs mathematical and statistical models to identify and establish patterns in massive data [17]. The potential of machine learning has been demonstrated in a variety of fields, including cancer diagnosis [18], genetics and genome sequence analysis [19], textual data classification [20], face recognition [21], and affect analysis [22,23,24].

Network traffic contains valuable information utilised for identifying potential intrusions. A feature-extraction process may extract a large number of features from the traffic that can be employed by a machine learning algorithm to build a model. However, not all features contribute to the decision-making process equally. Furthermore, they can impede classifier detection speed and potentially impact accuracy, false positives, etc. Therefore, feature selection is conducted to choose features that not only reduce model complexity but also enhance detection performance.

A feature selection (FS) process can be classified into three types: (i) filter, (ii) wrapper, and (iii) embedding [25]. Filter-based feature-selection methods use statistical and information theory techniques to identify robust features. However, these methods do not consider how the selected sub-features affect the chosen classifier [26]. To address this limitation, wrapper-based approaches utilise machine learning algorithms as fitness functions to find the best features [27]. Alternatively, embedded methods [28] offer a faster convergence compared to wrapper methods by integrating feature selection with the learning process.

In this paper, we propose a new wrapper-based feature-selection method. This new feature-selection algorithm is based on (i) Cellular Automata, (ii) Tabu Search, and (iii) Random Forest. Cellular Automata (CA) and Tabu Search (TS) are used as search strategy, while Random Forest (RF) works as a predictor function or learning process. The fitness of the solution is calculated by using Equation (3). A simulation shows that CAT-S algorithm shows promising results in achieving high accuracy, lowering the false positive rate, and reducing the number of features.

The rest of this paper is organised as follows. Section 2 presents the literature review, covering intrusion datasets for IoT networks and various dimension-reduction approaches used in network intrusion detection. In Section 3, we introduce our proposed CAT-S metaheuristics-based algorithm. In Section 4, we briefly provide insight into the TON_IoT dataset that is used in the study. In Section 5, we introduce a working example for understanding the operation of the CAT-S algorithm. In Section 6, we describe the environment designed to test the CAT-S algorithm. In Section 7, we present the results followed by the conclusion.

## 2. Literature Review

Datasets, feature selection, and ML classifiers are fundamental components in the realm of machine learning. They play pivotal roles in the development of predictive models and data-driven solutions. In this section, we provide a comprehensive overview of the datasets available for IoT networks, delve into various types of feature-selection techniques, and explore the related studies involving machine learning techniques employed in network intrusion-detection systems specifically designed for IoT networks.

### 2.1. Intrusion Datasets for IoT Networks

In general, intrusions are classified into two main types: (i) network-based intrusion and (ii) host-based intrusion [29]. Network-based intrusion datasets encompass network traffic captured over a specific time frame, containing both normal and malicious flow patterns. In contrast, host-based intrusion datasets focus on the activities of individual hosts or devices. This research centres around network intrusion detection and classification within IoT networks. As a result, in this section, we introduce IoT datasets designed for network intrusion-detection systems (NIDSs).

In the past two decades, numerous datasets [30] (e.g., DARPA 98-99, KDDCup99, NSL-KDD, DEFCON-10, Sperotto, MAWI Dataset, UNB ISCX, CTU-13, UNSW-NB15, UGR’16, and CICIDS 2017) have been published for network intrusion-detection systems (NIDSs). However, a similarity among these datasets is their focus on traditional networks. IoT networks present distinct challenges, featuring resource-constrained nodes with limited computational, battery, and storage capacities [31]. Consequently, they support lightweight operating systems, applications, network protocols, and APIs. Moreover, IoT network attack vectors differ significantly from those of legacy networks [32].

Hence, an imperative requirement arises for a proficient intrusion detection dataset tailored specifically to IoT networks. This dataset should encompass two key elements: (i) the inclusion of up-to-date attacks relevant to IoT networks and (ii) the incorporation of representative network traffic. In Table 1, we provide a summary of all pertinent IoT datasets published for network intrusion detection research. Table 1 includes dataset names, publication years, and the types of various attacks incorporated within each dataset.

### 2.2. Dimension-Reduction Techniques for Network Intrusion-Detection Systems (NIDSs)

In intrusion detection datasets, dealing with high-dimensional data is a common challenge. Dimension-reduction techniques are vital to combat the “curse of dimension”, benefiting both classification accuracy and computational efficiency. Existing methods include feature transformation and feature selection [33]. Feature-transformation techniques like extraction and construction reduce dimensionality, while feature selection aims to identify the most relevant features, enhancing classifier performance by eliminating redundancy and irrelevance. In the following section, we will briefly discuss the prevalent feature-selection approaches, which are widely favoured over feature transformation techniques today.

#### 2.2.1. Filter Approach

To select features, filter-based approaches utilise the “structural” characteristic of the data. They are independent of all learning algorithms, unlike wrapper approaches. They employ a number of feature ranking approaches to identify the real significance of features and whether or not to retain them. Rank or grade is computed using a variety of methodologies, including dataset statistical characteristics, entropy, and Laplacian scoring [34,35]. Filter algorithms are typically less computationally intensive than wrapper or embedded strategies; however, a major downside of filter-based techniques is that they are only appropriate for independent variables.

#### 2.2.2. Wrapper Approach

Wrapper-based feature-selection approaches consist of three parts: (i) a search strategy, (ii) a predictor function (learning process), and (iii) an evaluation or fitness function [29]. The search strategy selects the subset of features to be evaluated. The predictor approach utilises any classifier to assess the quality of the specified features to the objective or fitness function. The performance of the wrapper method is superior to that of filter-based selection approaches despite the fact that it requires more time than the filter method.

#### 2.2.3. Embedded Approach

Embedded approaches combine the positive characteristics of filter and wrapper methods. It actually introduces a relationship between the feature search strategy and the learning process (predictor function). Embedded approaches use a learning algorithm’s own variable-selection mechanism to choose and classify features simultaneously. Compared to the wrapper technique, it reaches convergence quickly and finds an optimal solution. Secondly, embedded approaches contribute to the reduction in overfitting or variance of a model by increasing its bias by penalising complexity.

### 2.3. Machine Learning-Based IDS Techniques for IoT Networks

This section describes the state-of-the-art machine learning and AI-based techniques used in intrusion detection for IoT networks. For this subject, we studied recent papers published in well-known journals since 2018 in this domain. In this regard, important findings from the papers were extracted, evaluated, and formalised to present in a table. A summary of the literature studied is presented in Table 2. The study was centred around the dataset and classifiers used in the research, as well as any feature-selection approaches being practised, followed by critical findings.

## 3. Hybrid Metahueristics-Based Feature Selection Method

In this section, we present our innovative feature-selection technique, CAT-S, designed specifically for IoT network intrusion detection. CAT-S is a hybrid wrapper-based feature-selection method that is based on (i) Cellular Automata (CA) [43], (ii) a Tabu metaheuristics search algorithm, and Random Forest (RF). Like Tabu Search, CAT-S is also a single-solution-based iterative algorithm. This means that it focuses on iteratively improving a single solution (feature subset). By combining the strengths of Cellular Automata, Tabu Search, and Random Forest, CAT-S aims to provide an effective feature-selection technique that enhances the accuracy and efficiency of IoT network intrusion detection.

### 3.1. Cellular Automata (CA)—Basics

A cellular automaton (CA) is a collection of cells organised in a predetermined grid so that each cell changes state as a function of time according to a given set of rules that are influenced by the states of adjacent cells. In the early 1960s, J. Von Neumann and Stan Ulam introduced the notion of Cellular Automata. Nonetheless, it remained inferior, and little research was performed until Wolfram, R. produced the massive book titled “A New Kind of Science”. Cellular Automata have drawn scientists from several fields. The popularity of CA is due to their simplicity and vast modelling capability for complex systems.

**Figure 2:**
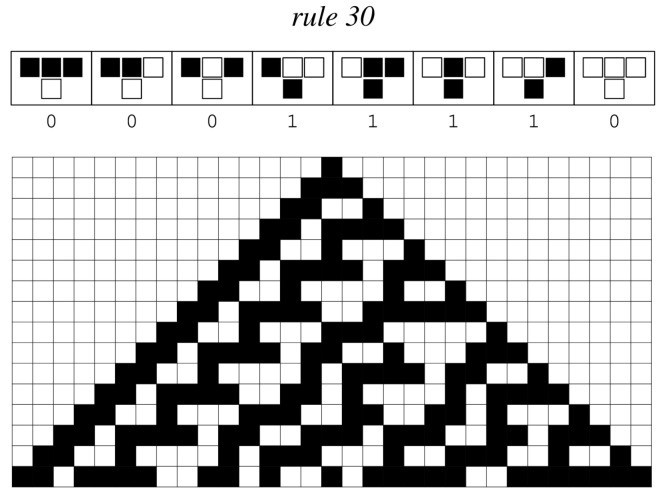
Rule 30 is one of the simple rules developed by Stephen Wolfram for Cellular Automata. It determines the next colour in a cell based on its current colour and the colours of its neighbours. Its rule results are encoded as 30 = 00011110 in binary format.

CA can be considered as a simplified model of a spatially extensive, decentralised system composed of a number of “cells” (Figure 2). Each individual cell maintains a distinct state that varies over time based on the states of neighbouring cells and the transaction rules. Despite its simplicity, the dynamics of CA are potentially quite rich when iterated multiple times; they vary from attractive stable configurations to spatio-temporal chaotic aspects and pseudo-random creation capabilities. These qualities allow for the possibility of surpassing local optimums when solving engineering challenges.

### 3.2. Tabu Search (TS)

Tabu Search [44] is a form of local neighbourhood search. Each viable solution has a collection of neighbours, denoted by N(S)⊆Ω, where Ω is a set of feasible solutions. A solution $S^{\prime }\subseteq N\left(S\right)$ can be achieved by performing a “move” from *S* to $S^{\prime }$. TS goes from a solution to its best admissible neighbour, even if doing so degrades the objective or fitness function. This is the non-greedy behaviour of Tabu Search, which is also advantageous for avoiding local optima traps.

To minimise cycling and trapping into local minima, recently investigated solutions are declared “prohibited” or “tabu”. These recently viewed solutions are maintained in a tabu list for a certain number of iterations. Each time a move is made, the tabu list is examined first. If a move is in the tabu list, the algorithm discards it and advances to the next iteration. When specific requirements (aspiration criteria or level) are met, the tabu status of a given solution might be overturned. An aspiration criterion is a mechanism used to temporarily override the tabu list “rule”. If the cost of the forbidden move is less than the aspiration level, then the tabu rule will be overridden and the move will be accepted.

### 3.3. Random Forest (RF)

Random Forest is essentially a tree-based classifier that generates a random number of decision trees. The classification of an input vector is decided by the ensemble’s predominant classification. Because it employs many ensemble techniques, it is also classified as an ensemble classifier [45]. RF is capable of classification and regression. In general, the performance of RF classifiers is superior to that of decision trees based on unseen data [46].

### 3.4. Fitness Function

In a wrapper-based feature-selection method, as discussed in Section 1, three fundamental components play a vital role in the selection process. The third significant component is the fitness or evaluation function, which is also referred to as the cost function or objective function.

In this context, the fitness function plays a critical role in guiding the feature-selection process toward identifying an optimal subset of features. It serves as a quantitative measure that defines the objective or goal to be achieved during the search for the best feature subset. The function evaluates the quality or performance of each potential solution (subset of features) within the search space.

Mathematically, we can represent the fitness function as f:S→R, where *f* denotes the function itself, *S* represents the search space consisting of all feasible subsets of features, and *R* represents the set of real numbers. The output of the fitness function for a specific feature subset is a real-valued score that quantifies how well the corresponding subset performs with respect to the chosen objective.

By calculating the fitness scores for various feature subsets, the feature-selection algorithm can iteratively explore the search space and identify the subset of features that optimises the chosen objective or minimises the associated cost function. The ultimate goal is to find the most relevant and informative features that lead to improved performance or accuracy in the specific task or problem at hand, such as classification, regression, or clustering.

Equation (1) presents the fitness calculation method. The formulation of Equation (1) draws inspiration from the principles governing artificial neural networks, specifically from the notion of assigning weights to individual inputs that contribute to the network’s overall output.

In this context, the fitness calculation, denoted as f(cost), serves as an objective function that assesses the quality of each solution (x) within the search space. The objective function quantifies the solution’s suitability or effectiveness in achieving the desired outcome. It encompasses a set of weighted factors, akin to the weights assigned to inputs in artificial neural networks, which collectively influence the fitness score.

Mathematically, the fitness calculation is represented as :

(1) $$f\left(cost\right)=\Sigma_{i=1}^{n}x_{i}w_{i}$$

where $x_{i}$ represents the objective we want to optimise, and $w_{i}$ is weight associated with the objective.

Our primary objectives are threefold: (i) enhancing the classification accuracy, (ii) reducing the false positive rate (FPR), and (iii) minimising the number of features used. However, accomplishing these goals simultaneously gives rise to a multi-objective optimisation problem with conflicting objectives, as improving accuracy often leads to an increase in FPR. To address this issue, we have transformed the conflicting objectives into non-conflicting ones using Equation (2). This equation allows us to calculate the classification error in terms of accuracy

(2) Error(e)=100−Accuracy

Equation (1) serves as a generic expression in this context. To cater to our specific objectives, we have derived a customised and expanded form of this equation, denoted as Equation (3). In Equation (3), the variables $x_{i}$ have been replaced with the actual objectives that we aim to optimise. Specifically, $x_{1}$ has been substituted with e, representing the classification error; $x_{2}$ with n, signifying the number of features; and $x_{3}$ with fpr, representing the weighted false positive rate. Our objective in this study is to (i) minimise the classification error (e), (ii) reduce the number of features in the feature vector, and (iii) lower the false positive rate. Therefore, this is a multiobjective minimisation problem, and our objective is to minimise the cost.

(3) $$f\left(cost\right)=w_{1}*e+w_{2}*n+w_{3}*fpr$$

In Equation (3) $w_{1}$ = 0.333, $w_{2}$ = 0.333 , $w_{3}$ = 0.333. Equal weights are assigned to each objective to avoid any influence.

### 3.5. Cellular Automata (CA)-Based Tabu Search (TS) Feature-Selection Algorithm (CAT-S)

**Figure 3:**
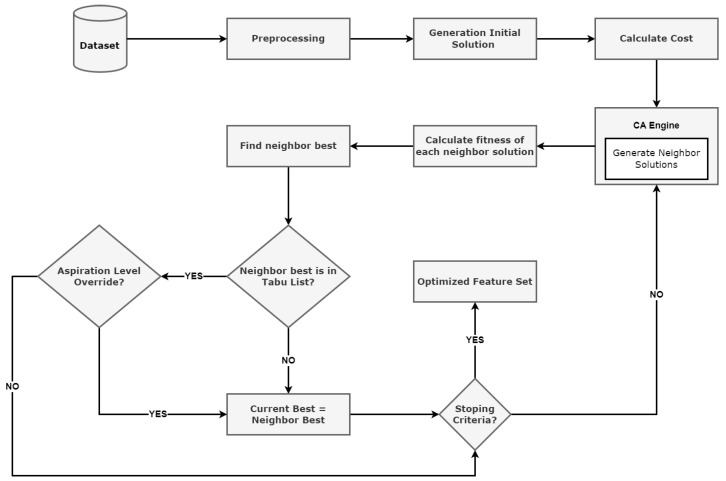
Flowchart presenting CAT-S proposed feature-selection algorithm.

In this section, we present the step-by-step working of our proposed CAT-S feature-selection algorithm. The proposed feature-selection algorithm is demonstrated in Figure 3.

#### 3.5.1. Data Preprocessing

Data preprocessing is a very important step that is performed during a machine learning process. In the preprocessing phase, various operations on the dataset are performed, e.g., (i) handling of missing values, (ii) encoding of categorical data, and (iii) data scaling, normalisation, standardisation, etc.

#### 3.5.2. Generating an Initial Solution and Calculating the Fitness

After the preprocessing of data is completed, the fitness of the initial feature vector is evaluated using Equation (3). The initial feature vector is also known as the initial solution. The initial solution is created by randomly selecting the features from the feature vector. The fitness of the initial feature vector and subsequent feature vectors is calculated by using the following steps.

1. The Random Forest classifier is used to find the accuracy, detection error (e), and false positive rate (fpr).
2. Error (e), number of features (n) in the feature vector, and (fpr) are input into Equation (3) to calculate the fitness.

The detail of the entire process is presented in Section 5.

#### 3.5.3. CA Engine—Generate Neighbour Solutions

After calculating the fitness of the initial solution (feature vector), the Cellular Automata engine is used to generate new solutions in each iteration. These possible solutions are known as candidate solutions or neighbour solutions. We generated five neighbour solutions in each iteration.

#### 3.5.4. Calculating the Fitness of Each Neighbour Solution

When the neighbour solutions are generated via the CA engine, the fitness (cost) of each neighbour solution is calculated by using Equation (3). Among the five neighbour solutions, the one with the lowest cost is selected as the best neighbour.

#### 3.5.5. Tabu List Lookup

The best neighbour selected in the previous step is checked in the Tabu List (TL). If the best neighbour solution is already present in the Tabu List, the solution will be set to the hold state until the aspiration level (AL) is checked.

#### 3.5.6. Aspiration Level Checking

As discussed in Section 3.2, if the cost of a forbidden move is less than the aspiration level, then the tabu rule will be overridden, and the move will be accepted. The aspiration level is a hyperparameter that is used to optimise the intensification and diversification strategies. In Table 3, we present the CAT-S algorithm hyperparameters.

#### 3.5.7. Accepting the Best Neighbour

In both cases, i.e., (i) if the neighbour best is not in the Tabu List or (ii) if the cost of the move is less than the aspiration level, then the move will be accepted. Please refer to the Section 5 for details.

#### 3.5.8. Stopping Criteria

The simulation will continue until the maximum iterations are not reached.

## 4. Dataset Details (TON_IoT)

In this section, we briefly discuss the details of the TON_IoT dataset and our simulation environment. The TON_IoT datasets represent a new generation of datasets focused on evaluating AI-enabled cybersecurity applications in the realm of IoT and IIoT (Industrial Internet of Things) networks. These datasets are composed of diverse data sources, including telemetry data from IoT and IIoT sensors, operating systems data from Windows 7 and 10, as well as Ubuntu 14.04 and 18.04 LTS and network traffic data.

The datasets were collected from a large-scale and realistic testbed network at the IoT Lab of the UNSW Canberra Cyber, School of Engineering and Information Technology (SEIT), UNSW Canberra. The testbed encompasses properties of Software-Defined Networking (SDN), Network Function Virtualisation (NFV), and Service Orchestration (SO), enabling communication between edge, fog, and cloud layers. The dataset includes nine different types of attacks, which are briefly presented below. It provides forty-six features/attributes (that include class label and attack category), which are collected from the network pcap files.

1. **Scanning attack:** This attack is alternatively known as a reconnaissance or probing attack, and it represents the initial phase in the cyber kill chain model or penetration testing. The primary objective of this attack is to gather information about the target systems, which involves identifying active IP addresses and open ports within the testbed network.
2. **Denial of Service (DoS) attack:** DoS refers to the act of flooding a network or IoT/IIoT services with fake requests in an attempt to disrupt or corrupt their resources.
3. **Distributed Denial of Service (DDoS) attack:** A DDoS is a sophisticated cyber attack that overwhelms a target with an enormous volume of fake requests from multiple sources simultaneously. The goal of DDoS attacks is to render a website or online service inaccessible to legitimate users, causing disruption and downtime. Perpetrators use networks of compromised devices (botnets) to orchestrate DDoS attacks, making them difficult to mitigate.
4. **Ransomware attack:** A ransomware attack is a type of malicious cyber attack where hackers encrypt the victim’s data and demand a ransom in exchange for the decryption key. Once infected, users are denied access to their files until the ransom is paid, posing significant risks to data privacy and business operations. Ransomware attacks are typically delivered through phishing emails, malicious downloads, or exploiting software vulnerabilities.
5. **Backdoor attack:** A backdoor attack is a stealthy and unauthorised method used by hackers to gain access to a computer system or network. It involves exploiting vulnerabilities to create hidden entry points, allowing attackers to bypass normal authentication measures. Backdoor attacks can result in unauthorised access, data breaches, and compromised system security.
6. **Injection attack:** An injection attack is a form of cyber attack where malicious code or commands are inserted into an application or system. These attacks exploit vulnerabilities, such as SQL injection, to manipulate the behaviour of the target and potentially gain unauthorised access or compromise data. Injection attacks pose significant risks to web applications, databases, and other software systems susceptible to user input manipulation.
7. **Cross-site Scripting (XSS) attack:** Cross-site scripting (XSS) is a type of cyber attack that allows attackers to inject malicious scripts into web pages viewed by other users. These scripts can then be executed in the context of the victim’s browser, stealing sensitive information or performing unauthorised actions on behalf of the user. XSS attacks pose a serious threat to web applications and can lead to the theft of user credentials, session hijacking, and other security breaches.
8. **Password cracking attack:** A password cracking attack is a cybersecurity technique aimed at gaining unauthorised access to user accounts by systematically guessing or decrypting passwords. Attackers use various methods such as brute force, dictionary attacks, or rainbow tables to crack weak or poorly protected passwords. Once successful, password cracking allows attackers to impersonate users, potentially leading to data breaches and compromising sensitive information.
9. **Man-In-The-Middle (MITM) attack:** A Man-in-the-Middle (MITM) attack is a cyber attack where an unauthorised actor intercepts and relays communications between two parties without their knowledge. During the attack, the attacker can eavesdrop, modify, or inject malicious content into the communication, potentially stealing sensitive information or gaining unauthorised access. MITM attacks pose significant risks to data privacy, online transactions, and the integrity of communication channels.

## 5. CAT-S Working Example

In this section, we present the working example for the proposed CAT-S feature-selection algorithm. When dataset preprocessing is completed, the initial feature vector is encoded.

### 5.1. Binary Encoding and Initial Solution

**Figure 4:**
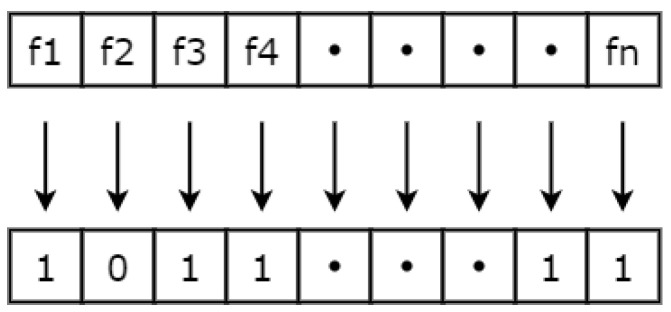
Feature vector encoding by using a binary bit pattern. A zero indicates that the feature is not selected, and a one indicates that the feature is selected for the study.

After the preprocessing phase, a unique *random* pattern of zeros and ones is generated, which is known as the initial solution. Each feature in the feature vector is assigned a binary value of 0 or 1. A binary one/zero in the bit pattern is used to indicate that the corresponding feature in the feature vector will be included (1) or excluded (0) from the current iteration of the study. A sample solution is presented in the Figure 4.

### 5.2. Calculate Cost of Initial Solution

After binary encoding of the feature vector, the cost of the initial solution is calculated by using Equation (3). This is the initial cost of the solution that is used to compare with the cost of neighbour solutions.

### 5.3. Generate Neighbour Solutions

**Figure 5:**
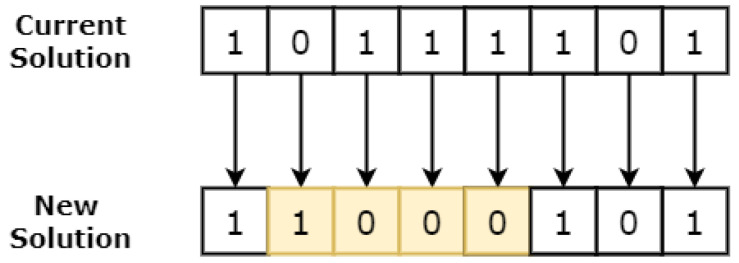
This figure shows how a new solution is generated from the existing solution. For each cell, CA rule 30 is applied; e.g., for the first cell 010, rule 30 will yield 1, and for the second cell 101, rule 30 will yield 1. The highlighted cells represents flipped cells.

The generated initial solution in the form of a bit vector is passed to the CA engine to generate neighbour solutions. The neighbour solutions are generated with the help of the CA rule 30. Rule 30 is an elementary cellular automaton that operates in a one-dimensional grid of cells, with each cell having two possible states: black (1) or white (0). It is characterised by its binary rule representation, which is 00011110 in binary or 30 in decimal. To compute the state of each cell in the next generation, rule 30 considers the current state of the cell and its immediate neighbours (the cell to the left and the cell to the right). It then looks up the corresponding pattern in its binary rule representation. Rule 30 looks at the three-cell neighbourhood in the current generation and determines the state of the center cell in the next generation based on the pattern formed by these three cells. There are a total of eight possible three-cell patterns ($2^{3}$), and rule 30 specifies what the centre cell’s state should be for each of these patterns. The process is repeated for each cell in the solution, generating the next generation of cells based on the rule and the previous generation as demonstrated in Figure 5. to find the next state of a cell.

### 5.4. Calculating the Fitness of Each Neighbour Solution

In each iteration, five neighbour solutions are generated, and their fitness is also calculated using Equation (3). Among all the generated neighbour solutions, one best solution is selected with the minimum cost, which is known as the neighbour best.

### 5.5. Tabu List Lookup

When a new neighbour is selected, that move is held in the Tabu List. The size of the Tabu List is set to 7, which means it keeps the last seven moves only, and in the next iteration, the oldest move is discarded. The purpose of the Tabu List is to prevent the algorithm from taking the same move and getting stuck in local minima.

### 5.6. Aspiration Level Checking

As discussed in Section 3.5.6, if the cost of the forbidden move (new solution) is less than the aspiration level, then the tabu rule will be overridden, and the move will be accepted.

The CAT-S algorithm continues till the stopping criteria are not reached.

## 6. Testing Configuration

In this section, we present the experimental methods employed for implementing the proposed algorithm and evaluating its performance. Various tools and languages commonly found in the literature were considered for implementing and evaluating the proposed IDS. These include Matlab, Python, C++, Weka tool, etc. Among these options, Weka emerged as the most user-friendly tool, offering support for several well-known algorithms. However, it suffered from a notable drawback in terms of processing time. To overcome this limitation, we built a custom-designed environment for implementing and testing our feature-selection algorithm.

The entire environment was built on Ubuntu Linux. We used the Python programming language for implementing Cellular Automata and Tabu Search. Since multiple candidate solutions are generated in each iteration, and their fitness needs to be computed, we employed ranger [47]—a fast implementation of Random Forests in C++. For each candidate solution, we created multiple threads that invoke ranger (*binary*), transferring control to ranger. Multiple threads are executed concurrently to achieve parallelism. This hybrid environment enabled us to conduct efficient and effective experiments while addressing processing time constraints.

Various metrics are employed to assess the performance of an intrusion-detection system (IDS). In the existing literature, the majority of research efforts in the field of intrusion detection have primarily concentrated on evaluating the accuracy and false positive rate (fpr). However, the specific focus of this research is to reduce the number of features incorporated into the IDS model, thereby reducing its overall complexity. Consequently, the number of features retained after the feature-selection process is identified as an additional crucial parameter to consider in this study.

To ensure the reliability and robustness of our experimental results, we conducted the experiment 100 times iteratively. This repetition aims to mitigate any potential statistical errors or fluctuations that could influence the outcome. By averaging the results obtained from these multiple runs, we enhanced the validity of our findings and achieved a more comprehensive understanding of the experiment’s outcomes.

## 7. Experiments and Results

**Figure 6:**
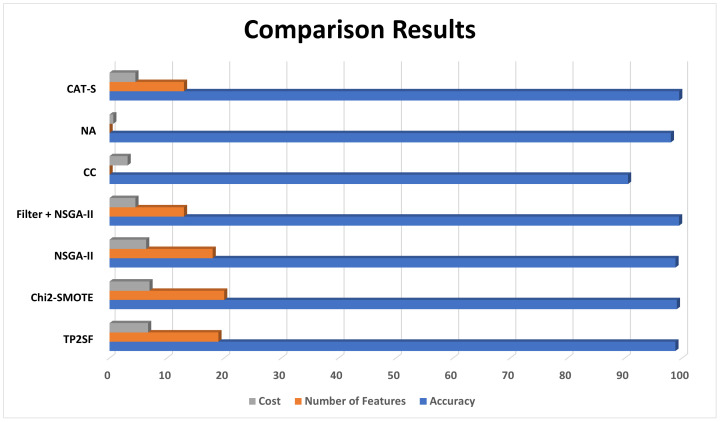
Comparison of the CAT-S algorithm with other state-of-the-art algorithms.

**Figure 7:**
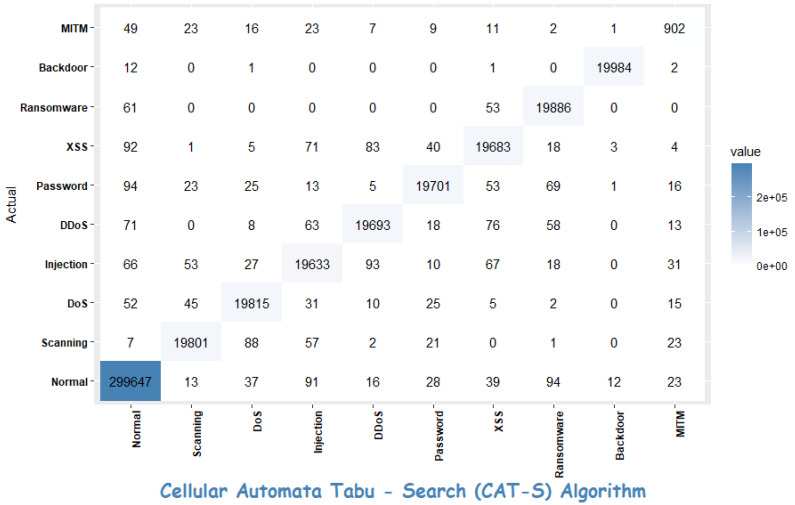
Confusion Matrix for CAT-S feature-selection algorithm.

In this section, we present the experimental analysis conducted to evaluate the effectiveness of the proposed CAT-S feature-selection algorithm, along with its corresponding results. Our evaluation involved a comparison with the state-of-the-art feature-selection techniques reported in recent studies. The assessment was performed on the ToN_IoT dataset. The comparison results are summarised in Table 4 and Figure 6, which includes key metrics such as the classification accuracy, the false positive rate, and the number of features selected. In addition to this, Figure 7 presents the confusion matrix of the proposed CAT-S feature-selection algorithm.

Figure 6 provides a comprehensive overview of the variables on which the evaluation was performed, including the accuracy, the false positive rate, and the number of selected features. It is worth noting that, in several recent papers, the false positive rate (FPR) and precision measure were not considered during the evaluation process, which shows a significant weakness in their proposition.

The simulation outcomes indicate that the CAT-S algorithm demonstrated superior performance in comparison to other state-of-the-art feature-selection techniques. CAT-S achieved improved attack detection accuracy around 95.5% while simultaneously reducing the number of features required for the IDS model. The CAT-S algorithm achieved higher accuracy and a lower false positive rate when the number of selected features reached 13. The false positive rate for the CAT-S algorithm reaches 0.004, which is considerably good. By reducing the number of features to 13, CAT-S has succeeded in reducing the number of features by over 72% while achieving higher accuracy and a better false positive rate.

A confusion matrix is a table or matrix used in machine learning and statistics to assess the performance of a classification model. It provides a summary of the predictions made by a model compared to the actual or true outcomes (ground truth) in a classification problem. A confusion matrix typically consists of four components:

**True Positives (TP):** The number of instances that were correctly predicted as positive by the model. In other words, these are the cases where the model correctly identified positive instances.

**True Negatives (TN):** The number of instances that were correctly predicted as negative by the model. These are the cases where the model correctly identified negative instances.

**False Positives (FP):** The number of instances that were predicted as positive by the model but were actually negative. These are also known as Type I errors or false alarms.

**False Negatives (FN):** The number of instances that were predicted as negative by the model but were actually positive. These are also known as Type II errors or missed detections.

In multiclass classification, the confusion matrix is an extension of the concept used in binary classification, but it involves more than two classes. The confusion matrix for multiclass classification provides a detailed summary of how a classification model performs across all the classes in the problem. As discussed, Figure 7 represents the confusion matrix of our proposed feature-selection algorithm. It has classes, including “Normal”, “Scanning”, “DoS”, “Injection”, “DDoS”, “Password”, “XSS”, “Ransomware”, “Backdoor”, and “MITM”.

Precision (also known as positive predictive value) is calculated as the number of correct positive predictions divided by the total number of positive predictions. The best precision is 1.0, whereas the worst is 0.0. The formula to calculate precision is presented in Equation (4):

(4) $$Precision=\frac{TP}{TP+FP}$$

Table 5 shows the precision calculated for each category separately.

### Critical Discussion

Our findings indicate that CAT-S demonstrates remarkable performance in terms of attack detection accuracy and feature reduction. The algorithm achieves an accuracy of 99.50%, outperforming the other techniques, which exhibit lower accuracy values. Moreover, CAT-S significantly reduces the number of features down to 13 while still maintaining excellent classification performance. Additionally, the false positive rate achieved by CAT-S is impressively low at 0.004, showcasing its ability to effectively distinguish between normal and malicious instances.

The CAT-S algorithm is based upon a hybrid metaheuristic approach, which has demonstrated its efficacy in searching for optimal solutions within extensively large search spaces. It has a relatively reduced computational burden compared to techniques rooted in machine learning or deep learning that require training over large datasets. This advantage can be attributed to the inherent capacity of metaheuristic algorithms to systematically explore the entirety of the search space, with the overarching objective of identifying the global optimum. In contrast, machine learning and deep learning models are susceptible to becoming ensnared within local optima. Below, we discuss the overall strengths and weaknesses identified during the study.

- The normal class has the number of highest true positives; however, it also has a significant number of false positives for some other classes (e.g., “Injection”, “DDoS”). The high false positives suggest that the classifier occasionally misclassifies instances as “Normal” when they belong to other classes.
- The scanning and DoS classes have a relatively high number of true positives and fewer false positives compared to some other classes. It indicates that the model performs relatively well in identifying instances of these classes.
- The injection class also has a reasonably high number of true positives but has a moderate number of false positives, particularly for the “Normal” class. This suggests that the model sometimes misclassifies “Injection” instances as “Normal”.
- The DDoS class has a high number of true positives and a relatively low number of false positives, indicating that the model performs well in identifying instances of this class.
- For other classes like Password, XSS, etc., they have varying numbers of true positives and false positives. The model’s performance in these classes may require further investigation and potentially fine-tuning.
- The precision values for the majority of the classes, such as “Normal”, “Scanning”, “DoS”, and “Password”, are very high, exceeding 0.98. This suggests that the model performs exceptionally well in correctly identifying instances of these classes and minimising false positives.
- The MITM class has moderately low precision as compared to other classes. This may be due to the smaller number of samples for the MITM class.

## 8. Conclusions

This research paper introduces a novel hybrid wrapper-based feature-selection algorithm that integrates Cellular Automata (CA) with the Tabu Search metaheuristic technique. The primary objective of this algorithm is to minimise the number of features, maximise the classification accuracy and lower the false postive rate (for), specifically for IoT networks. The proposed approach consists of three main steps. Firstly, various preprocessing operations are applied to transform the dataset into a suitable format for machine learning tasks. Secondly, the CAT-S feature-selection algorithm, which combines CA and TS, is formulated and implemented. In the third step, the performance of CAT-S is evaluated by comparing it with recently published state-of-the-art approaches. To assess the effectiveness of the algorithm, experiments are conducted on the TON_IoT dataset, specifically designed for IoT networks. The simulation results indicate that the CAT-S algorithm significantly improves attack-detection accuracy while simultaneously reducing the false positive rate (fpr) and the number of features by more than 70%.

## Figures and Tables

**Table 1:** IoT Networks Datasets.

Year	Dataset	Attacks
2018	N-BaIot	Botnet (Mirai and BASHLITE)
2019	Bot-IoT	DoS/DDoS, Botnet, Information theft (data exfiltration,
		keylogging), Reconnaissance (OS fingerprint, service scan)
2019	UNSW-IoT	DDos, MITM
2019	IoT Network Intrusion Dataset	DDoS, Botnet, MITM, Scanning
2020	TON IoT	DDoS, Ransomware, Backdoor, Data Injection
		XSS, Password Cracking attack, MITM
2020	IoTID20	DDoS, Botnet, MITM, Scanning

**Table 2:** Comparison of Related Work: This table presents a survey of notable articles published since 2018.

Year	Ref.	Dataset	Classifiers /Technique	FeatureSelection	Critical Comments
2018	[31]	NSL-KDD	Proposed a distributed Deep Learning (DL) model.	None	The authors claimed that the performance of the proposed distributed DL model is better than traditional machine learning systems; however, the comparison results are not presented. The authors used the NSL-KDD dataset, which was published in the year 2000 and was not designed to represent network traffic and attack vectors of current IoT systems.
2019	[36]	UNSW-NB15, NIMS botnet dataset with simulated sensors’ data	Proposed AdaBoost-based ensemble learning	Coefficient Correlation	Proposed the AdaBoost ensemble learning method by using three ML techniques. Decision Tree (DT), Naive Bayes (NB) and Artificial Neural Networks (ANNs). Comparison is performed on DNS and HTTP traffic. Comparison results showed that the proposed ensemble technique performed better than DT, NB, and ANN.
2019	[37]	Designed and deployed IoT testbed for data collection	They used nine different classifiers for testing. NB, BN, J48, Zero R, OneR, Simple Logistic, SVM, MLP, RF	Gain ratio, coefficient correlation	Proposed model comprised three-layer design to detect intrusion, i.e., (i) classifies the type of attack and profiles the normal behaviour of IoT appliances, (ii) identifies malicious packets, and (iii) classifies the type of the attack. The study is carried out in a custom design testbed built for evaluation.
2019	[38]	Collected traffic from the testbed	They studied seven different techniques SVM, KNN, NB, RF, DT, LR and ANN	Yes, chose features whose values change during attack phases compared to normal operation phases. Feature Ranking	The authors built a real-world testbed to conduct attacks and design an IDS. They performed backdoor, command injection and SQLi attacks. Results shows that Random Forest’s accuracy is highest among all classifiers.
2020	[39]	CICIDS 2017	Proposed DL-based technique Deep Belief Network (DBN) and compared with SVMIDS, RNNIDS, SNNIDS, FNNIDS	None	The authors compared DBN with other mentioned techniques. Simulation results show that DBN performed better than the other studied techniques.
2020	[40]	Built their own dataset by collecting logs from in house testbed	Passban—IDS	None	The authors designed and built an anomaly-based IDS for attack detection. They launched port scanning, http and ssh brute force, and syn flooding attacks The results are not compared with other approaches.
2021	[41]	BoT-IoT, IoT Network Intrusion, MQTT- IoT-IDS2020, and IoT-23	Proposed a novel intrusion detection model based on CNN by using transfer learning.	Recursive Feature Elimination (RFE)	Proposed CNN-based model for IDS. Transfer learning is used to implement binary and multiclass classification.
2021	[42]	IoTID20	CNN, LSTM and hybrid CNN-LSTM model	PSO	Comparison with state-of-the-art techniques proved that it has good performance

**Table 3:** CAT-S algorithm parameters.

Parameter	Value
Tabu List size	7
No. of neighbours in each iteration	5
Aspiration level	0.02
Stopping criteria	Max. number of iterations (set to 100)

**Table 4:** Comparison of CAT-S with state-of-the-art methods. Results show that CAT-S achieved higher accuracy and a lower false positive rate with a reduced no. of features.

Reference	Classifier	Feature-Selection Technique	Accuracy	FPR	Number of Features	Cost
Kumar et al. [48]	XG-Boost	TP2SF	98.84	NA	19	6.713 + Δfpr
Gad et al. [49]	XG-Boost	Chi2-SMOTE	99.10	NA	20	6.959 + Δfpr
Dey et al. [50]	SVM	NSGA-II	98.86	NA	18	6.373 + Δfpr
Dey et al. [50]	SVM	Filter + NSGA-II	99.48	NA	13	4.502 + Δfpr
Oseni et al. [51]	CNN	CC	90.55	NA	NA	3.146 + *n* + Δfpr
M Sarhan et al. [52]	Extra Trees	NA	98.05	NA	NA	0.649 + *n* + Δfpr
**CAT-S**	RF	CAT-S	99.50	0.004	13	4.496

**Table 5:** Precision of each attack type, calculated using Equation (4).

Type	Precision
Normal	0.998320845
Scanning	0.992083772
DoS	0.989661372
Injection	0.982534281
DDoS	0.989150635
Password	0.992393713
XSS	0.984740845
Ransomware	0.986996228
Backdoor	0.999150042
MITM	0.876579203

## Data Availability

Not applicable.
